# Supplementary material for: ALPHLARD: a Bayesian method for analyzing HLA genes from whole genome sequence data
Source: BMC Genomics. 2018 Nov 1;19:790. doi: 10.1186/s12864-018-5169-9 (PMC6211482; doi:10.1186/s12864-018-5169-9)
Supplement: Supplementary file 3 — Table S14. WGS-based HLA genotyping accuracy that indicates how many samples were fully correctly genotyped with ALPHLARD, OptiType, PHLAT, and HLA-VBSeq. (PDF 11 kb) [file 12864_2018_5169_MOESM3_ESM.pdf]

Table S14: WGS-based HLA genotyping accuracy that indicates how many samples were fully correctly genotyped with ALPHLARD, OptiType, PHLAT, and HLA-VBSeq. N/A indicates that the method does not support the HLA gene or the resolution.

		ALPHLARD	OptiType	PHLAT	HLA-VBSeq
HLA-A	1st	**100% (25/25)**	**100% (25/25)**	64.0% (16/25)	92.0% (23/25)
	2nd	**96.0% (24/25)**	**96.0% (24/25)**	36.0% (9/25)	72.0% (18/25)
	3rd	**96.0% (24/25)**	N/A	20.0% (5/25)	72.0% (18/25)
HLA-B	1st	**100% (24/24)**	79.2% (19/24)	66.7% (16/24)	79.2% (19/24)
	2nd	**100% (24/24)**	75.0% (18/24)	45.8% (11/24)	54.2% (13/24)
	3rd	**91.7% (22/24)**	N/A	25.0% (6/24)	50.0% (12/24)
HLA-C	1st	**100% (25/25)**	**100% (25/25)**	76.0% (19/25)	92.0% (23/25)
	2nd	**96.0% (24/25)**	92.0% (23/25)	44.0% (11/25)	40.0% (10/25)
	3rd	**96.0% (24/25)**	N/A	28.0% (7/25)	40.0% (10/25)
HLA-DPA1	1st	**100% (12/12)**	N/A	N/A	75.0% (9/12)
	2nd	**100% (12/12)**	N/A	N/A	75.0% (9/12)
	3rd	**100% (12/12)**	N/A	N/A	75.0% (9/12)
HLA-DPB1	1st	**100% (11/11)**	N/A	N/A	81.8% (9/11)
	2nd	**100% (11/11)**	N/A	N/A	81.8% (9/11)
	3rd	**100% (11/11)**	N/A	N/A	81.8% (9/11)
HLA-DQA1	1st	**100% (12/12)**	N/A	66.7% (8/12)	**100% (12/12)**
	2nd	**91.7% (11/12)**	N/A	50.0% (6/12)	**91.7% (11/12)**
	3rd	**91.7% (11/12)**	N/A	50.0% (6/12)	**91.7% (11/12)**
HLA-DQB1	1st	**100% (9/9)**	N/A	77.8% (7/9)	**100% (9/9)**
	2nd	**88.9% (8/9)**	N/A	44.4% (4/9)	77.8% (7/9)
	3rd	**88.9% (8/9)**	N/A	33.3% (3/9)	77.8% (7/9)
HLA-DRB1	1st	**100% (12/12)**	N/A	58.3% (7/12)	91.7% (11/12)
	2nd	**100% (12/12)**	N/A	33.3% (4/12)	41.7% (5/12)
	3rd	**100% (12/12)**	N/A	33.3% (4/12)	41.7% (5/12)
Total	1st	**100% (130/130)**	93.2% (69/74)	68.2% (73/107)	88.5% (115/130)
	2nd	**96.9% (126/130)**	87.8% (65/74)	42.1% (45/107)	63.1% (82/130)
	3rd	**95.4% (124/130)**	N/A	29.0% (31/107)	62.3% (81/130)
