# Supplementary material for: ALPHLARD: a Bayesian method for analyzing HLA genes from whole genome sequence data
Source: BMC Genomics. 2018 Nov 1;19:790. doi: 10.1186/s12864-018-5169-9 (PMC6211482; doi:10.1186/s12864-018-5169-9)
Supplement: Supplementary file 4 — Figures S1 and S2. Somatic point mutations in microsatellite-unstable colon cancer samples. (PDF 291 kb) [file 12864_2018_5169_MOESM4_ESM.pdf]

a

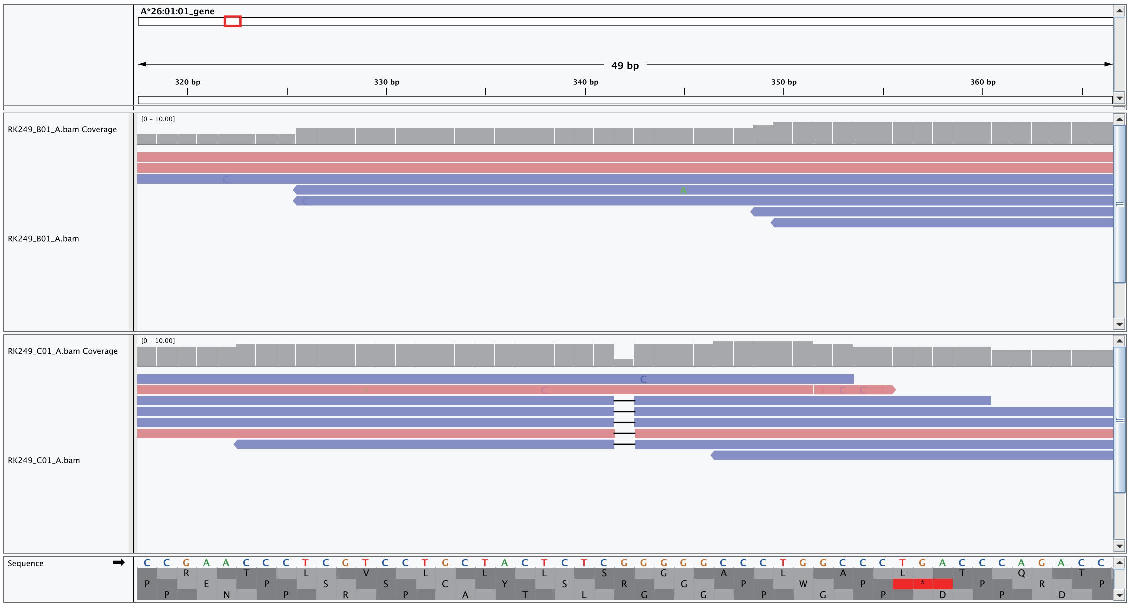

b

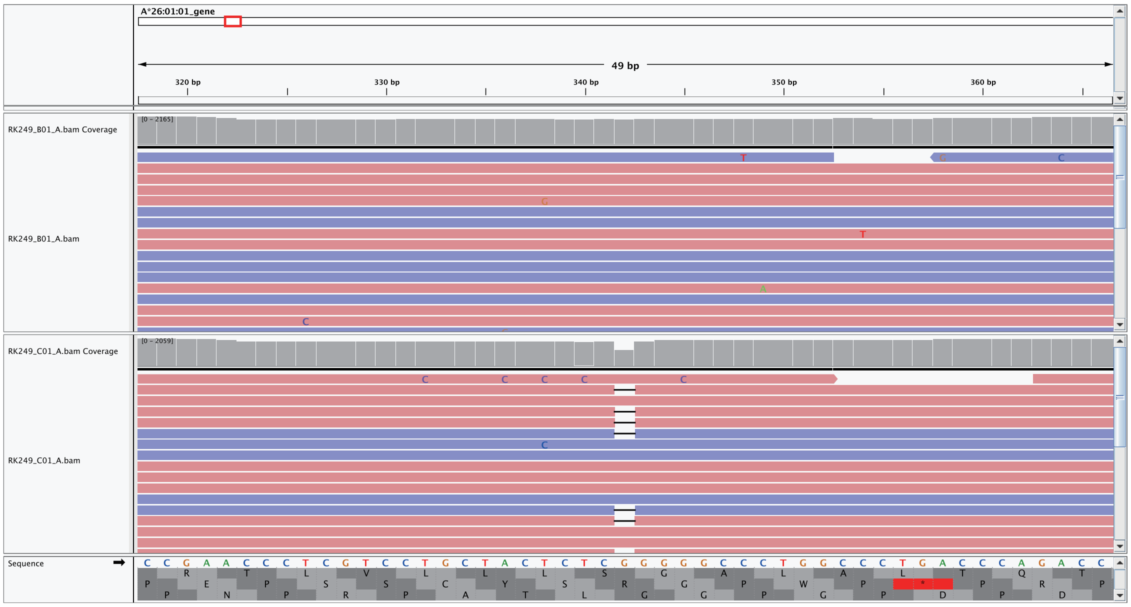

Figure S1: A single-base deletion in exon 1 of the HLA-A gene of patient RK249. IGV screenshots were taken at the position for the WGS data (upper) and the TruSight HLA Sequencing Panel data (lower). In each of the screenshots, the upper and lower tracks correspond to the normal and tumor samples, respectively.

a

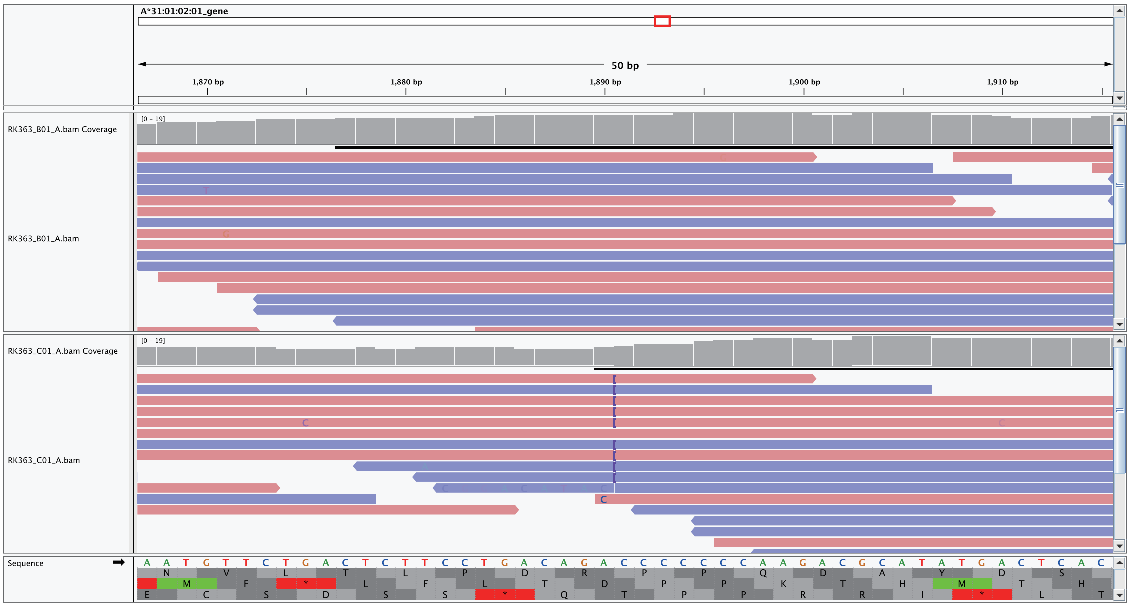

b

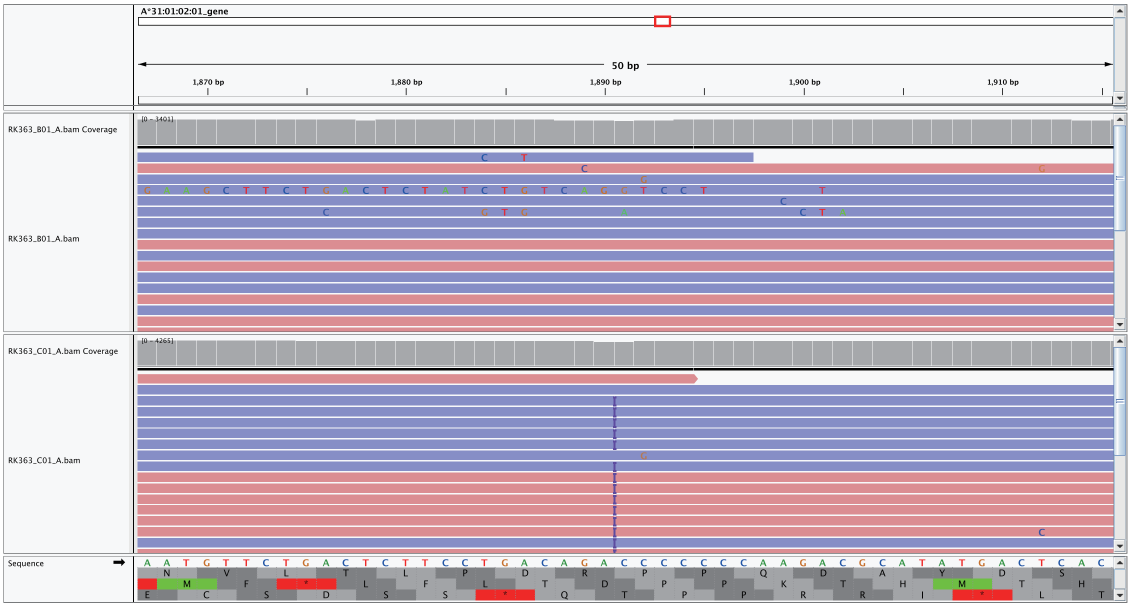

Figure S2: A single-base insertion in exon 4 of the HLA-A gene of patient RK363. IGV screenshots were taken at the position for the WGS data (upper) and the TruSight HLA Sequencing Panel data (lower). In each of the screenshots, the upper and lower tracks correspond to the normal and tumor samples, respectively.
