# Supplementary material for: ALPHLARD: a Bayesian method for analyzing HLA genes from whole genome sequence data
Source: BMC Genomics. 2018 Nov 1;19:790. doi: 10.1186/s12864-018-5169-9 (PMC6211482; doi:10.1186/s12864-018-5169-9)
Supplement: Supplementary file 5 — Figures S3-S7. Log odds ratios of the depths of heterozygous HLA SNPs in a liver cancer sample. (PDF 104 kb) [file 12864_2018_5169_MOESM5_ESM.pdf]

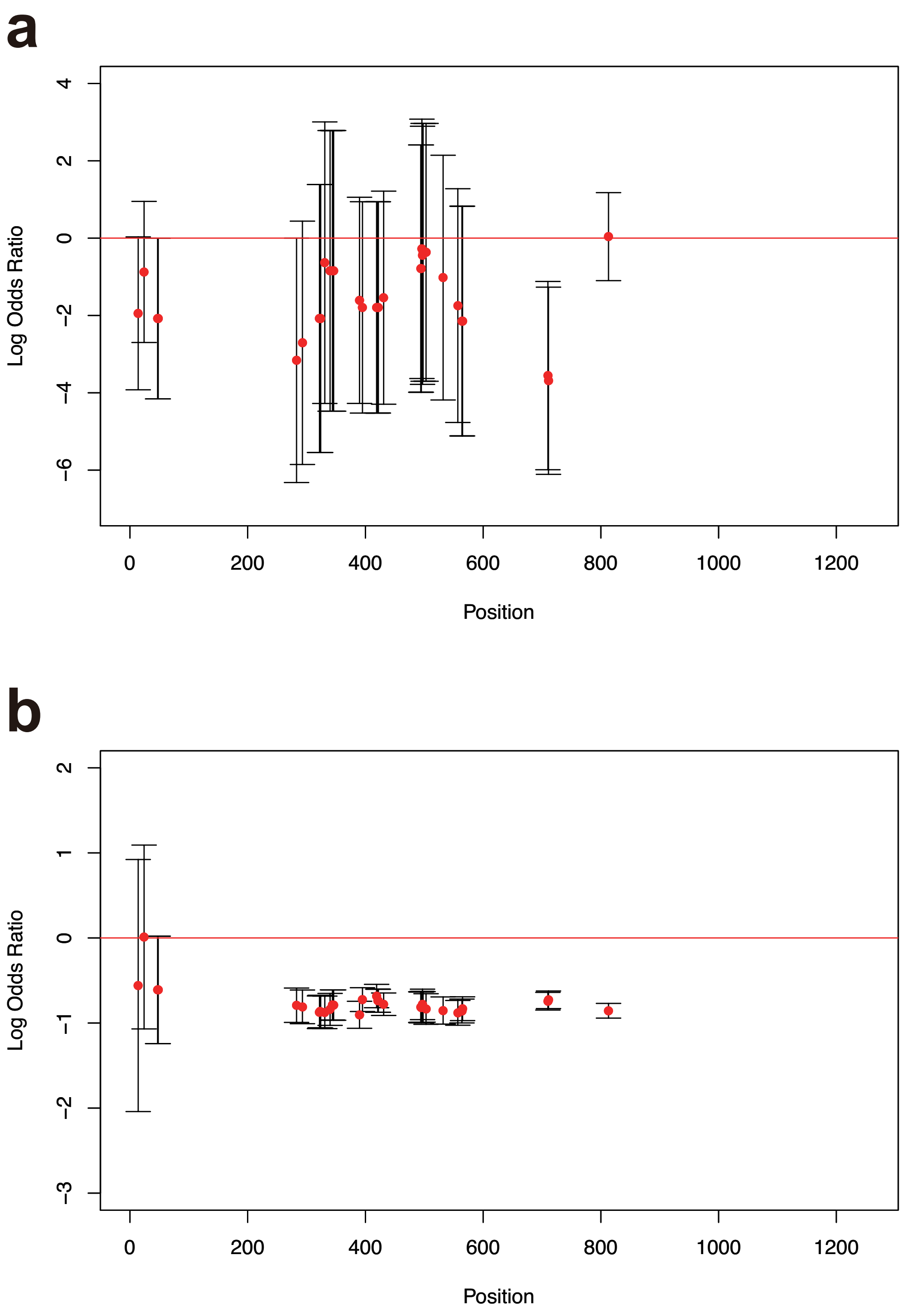

Figure S3: The log odds ratios of the depths at heterozygous SNP positions in the HLA-B gene of patient RK069. The log odds ratios were calculated for the WGS data (upper) and the TruSight HLA Sequencing Panel data (lower). These log odds ratios correspond to the relative quantities of observed B*35:01:01 SNPs in the tumor sample compared with the normal sample. The red dots indicate the mean values of the log odds ratios, and the vertical lines indicate the 95% confidence intervals.

a

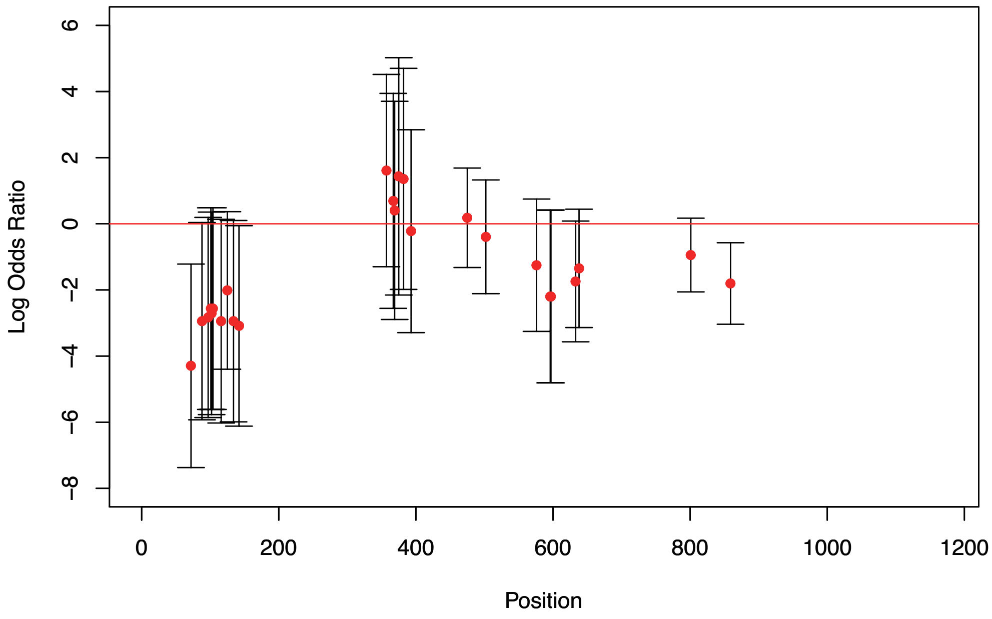

b

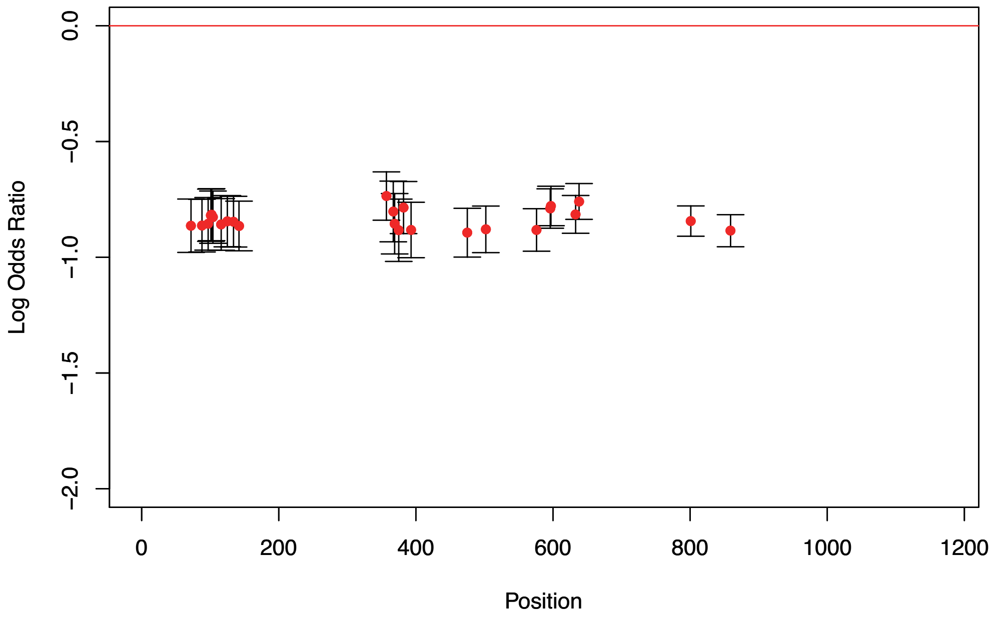

Figure S4: The log odds ratios of the depths at heterozygous SNP positions in the HLA-C gene of patient RK069. The log odds ratios were calculated for the WGS data (upper) and the TruSight HLA Sequencing Panel data (lower). These log odds ratios correspond to the relative quantities of observed C*03:03:01 SNPs in the tumor sample compared with the normal sample. The red dots indicate the mean values of the log odds ratios, and the vertical lines indicate the 95% confidence intervals.

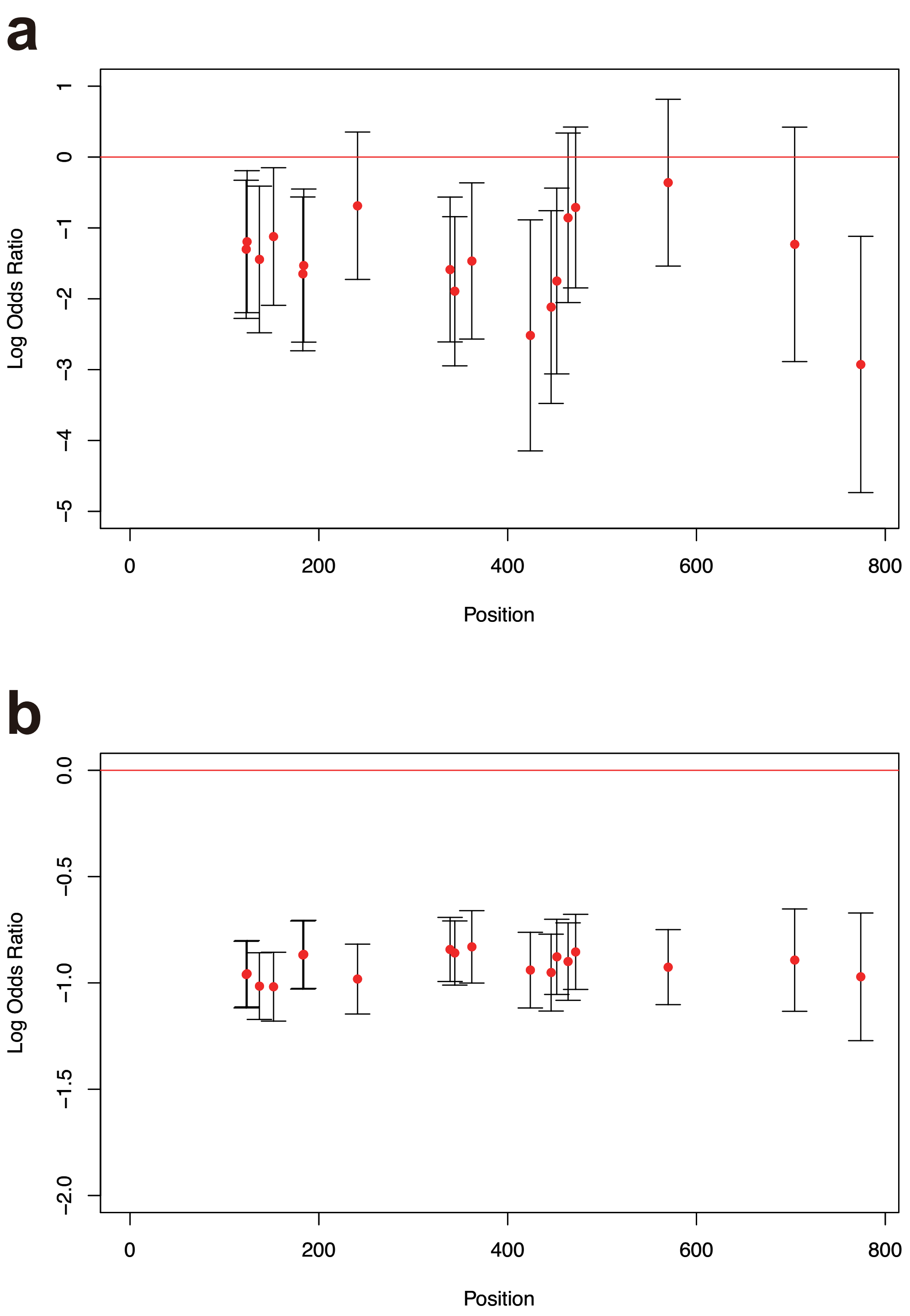

Figure S5: The log odds ratios of the depths at heterozygous SNP positions in the HLA-DPA1 gene of patient RK069. The log odds ratios were calculated for the WGS data (upper) and the TruSight HLA Sequencing Panel data (lower). These log odds ratios correspond to the relative quantities of observed DPA1*01:03:01 SNPs in the tumor sample compared with the normal sample. The red dots indicate the mean values of the log odds ratios, and the vertical lines indicate the 95% confidence intervals.

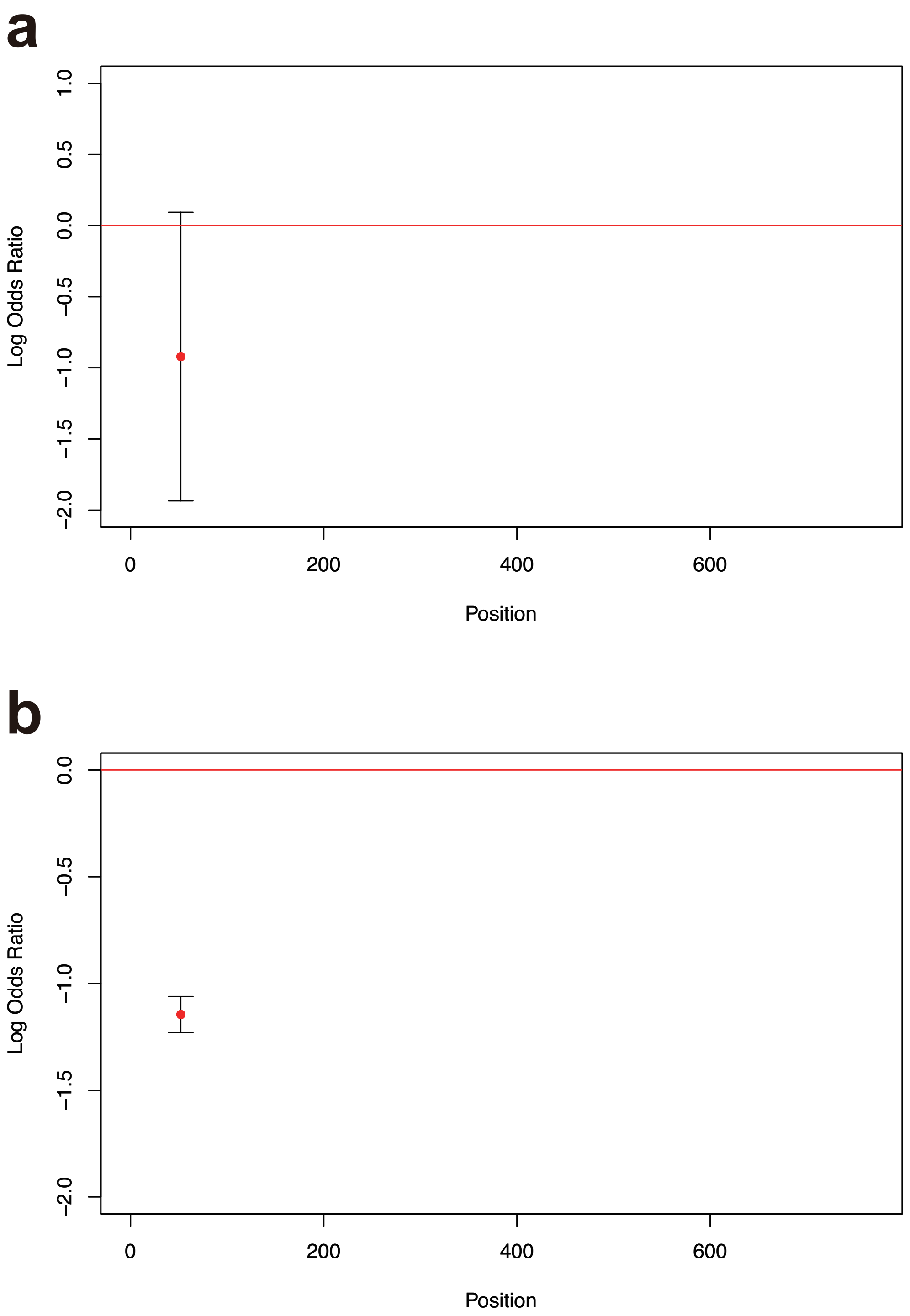

Figure S6: The log odds ratios of the depths at heterozygous SNP positions in the HLA-DQA1 gene of patient RK069. The log odds ratios were calculated for the WGS data (upper) and the TruSight HLA Sequencing Panel data (lower). These log odds ratios correspond to the relative quantities of observed DQA1*03:02 SNPs in the tumor sample compared with the normal sample. The red dots indicate the mean values of the log odds ratios, and the vertical lines indicate the 95% confidence intervals.

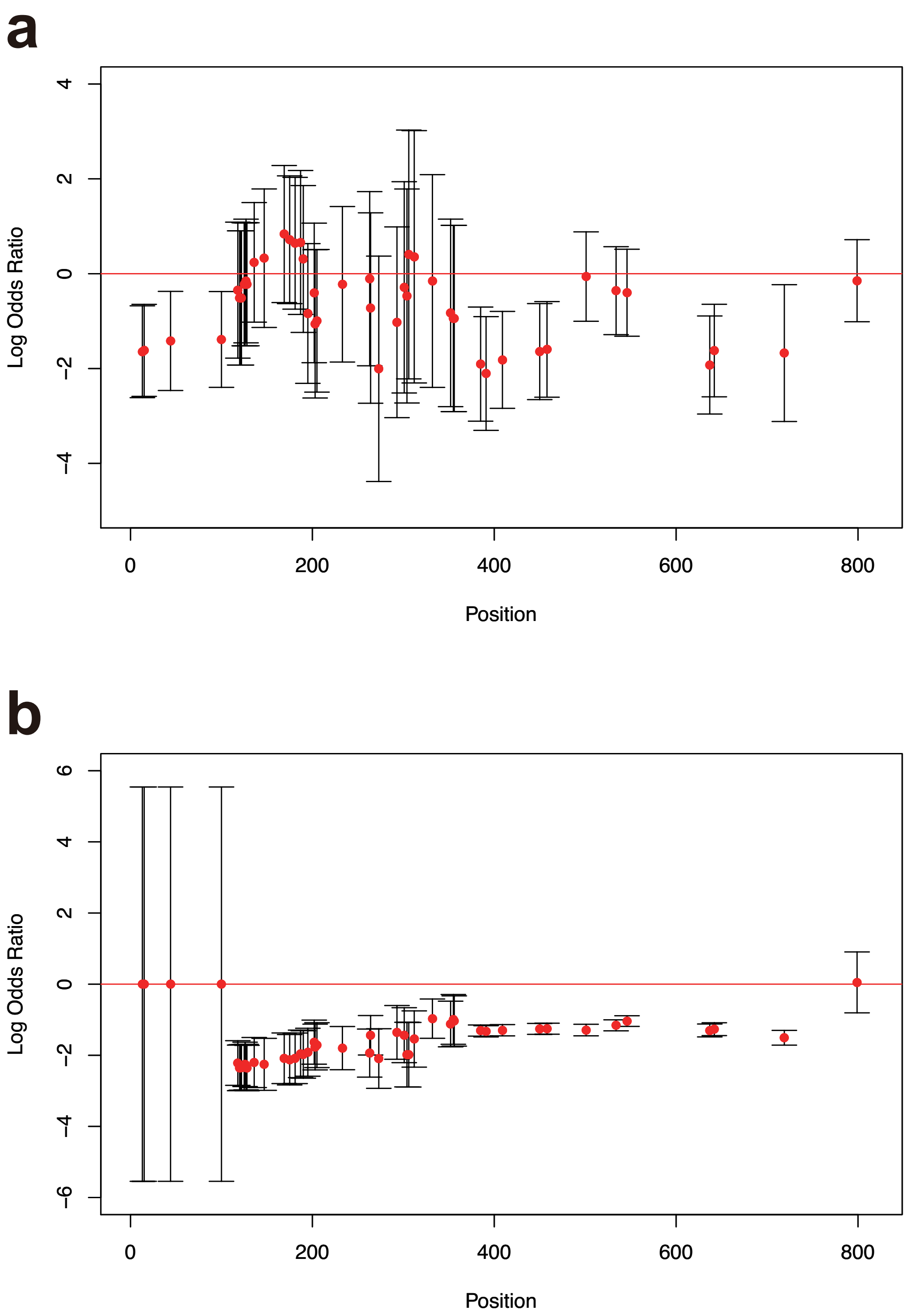

Figure S7: The log odds ratios of the depths at heterozygous SNP positions in the HLA-DRB1 gene of patient RK069. The log odds ratios were calculated for the WGS data (upper) and the TruSight HLA Sequencing Panel data (lower). These log odds ratios correspond to the relative quantities of observed DRB1*12:01:01 SNPs in the tumor sample compared with the normal sample. The red dots indicate the mean values of the log odds ratios, and the vertical lines indicate the 95% confidence intervals.
